# Impacts of Non-Lethal High-Temperature Stress on the Development and Reproductive Organs of *Bradysia odoriphaga*

**DOI:** 10.3390/insects13010074

**Published:** 2022-01-10

**Authors:** Jingrong Hu, Rudoviko Galileya Medison, Seng Zhang, Peifang Ma, Caihua Shi

**Affiliations:** 1 Department of Plant Protection, College of Agriculture, Yangtze University, Jingzhou 434025, China; hujingrong2017@126.com (J.H.); rudovikogalielya@126.com (R.G.M.); zhangsengzs@126.com (S.Z.); 2 Forewarning and Management of Agricultural and Forestry Pests, Hubei Engineering Technology Center, Yangtze University, Jingzhou 434025, China; 3 Henan Engineering Research Center of Chinese Chives, Pindingshan Academy of Agricultural Sciences, Pindingshan 467000, China; mapeifangma@126.com

**Keywords:** *Allium tuberosum*, chive gnats, emergence, fertility, sublethal

## Abstract

**Simple Summary:**

*Bradysia odoriphaga* is a soil-dwelling insect native to China, and its preferred host is Chinese chives. In this study, non-lethal high-temperature as an important limiting factor to affect the population and development of *B. odoriphaga* was assessed. Meanwhile, the physiological mechanism on non-lethal high-temperature to reduce the population was also identified. These would lay a key theoretical foundation for the future development of high-temperature products for environment friendly pest control strategies.

**Abstract:**

*Bradysia odoriphaga* is an agricultural pest in China’s vegetable industry. In this study, pupae and adults were exposed to various non-lethal high-temperatures. The results demonstrated a decreased rate of eclosion once the pupae were exposed to temperatures exceeding 37 °C for 1 h. No effect on the lifespan of unmated female adults was observed after exposure to temperature stress, while unmated male adult lifespan decreased (>37 °C for 2 h). The size of the testis and ovaries for unmated male and female adults decreased, as did the fecundity and egg hatching rate for mated females. Compared with the control group (25 °C), the testis size of unmated male adults decreased after high-temperature stress followed by recovery at 25 °C for 1 h, though the size of the ovaries of female adults did not change. Additionally, the size of the testis and ovaries for unmated male and female adults decreased following high-temperature stress and 24 h of recovery at 25 °C. High temperatures affected males more than females; 37 °C is the critical temperature to control the population of *B. odoriphaga*. These results lay the foundation for the future development of environmentally friendly high-temperature prevention and pest-control strategies.

## 1. Introduction

*Bradysia odoriphaga* Yang et Zhang (Diptera: Sciaridae) is commonly known as the chive maggot and consumes roots [1]. It has a wide range of hosts and can damage over thirty kinds of vegetables and fruits from seven families, as well as edible fungi [2,3]. These hosts include, but are not limited to, Welsh onion, garlic, lettuce, celery, Chinese cabbage, and Chinese chives (*Allium tuberosum* Rottler ex Spreng). If control measures are not used against *B. odoriphaga*, yields of *A. tuberosum* can be reduced by 50%. In severe cases, the seedlings can be destroyed, leading to near-total crop failure [4].

The methods currently used to prevent and control *B. odoriphaga* include releasing entomopathogenic nematodes [5,6], hanging black sticky boards [7], and adjusting cultivation patterns [8]. However, these methods are limited by high costs and poor efficacy. Vegetable farmers use root irrigation of overdose chemical pesticides [9], which has conferred resistance on *B. odoriphaga* and resulted in environmental pollution and food safety concerns [10]. Therefore, research on green control technology for *B. odoriphaga* would be critical for ensuring healthy and sustainable agricultural productivity.

While many environmental factors influence insect growth, reproduction, and population structure [11,12], temperature is one of the most important limiting factors [13]. High temperatures can impact insect survival, growth and development, lifespan, reproductive rate, sex ratio, gene expression, and heat shock protein synthesis [14]. For instance, extreme high-temperatures hinder the feeding of *Leucoptera coffeella* Guérin-Méneville (Lepidoptera: Lyonetiidae) [15], affect the egg laying of *Spodoptera exigua* Hübner (Lepidoptera: Noctuidae) [16]. *Bactrocera cucurbitae* Coquillet (Diptera: Tephritidae) adults will die when the temperatures exceed 51 °C [17]. In short, if the temperatures are higher than the optimum temperature range for development, the adaptability of insects will instantly fall with the temperature increase [18]. Recently, Shi et al. [19] also identified a negative relationship between the population of *B. odoriphaga* and non-lethal high-temperatures in nature. A similar study demonstrated that non-lethal high-temperature stress on *B. odoriphaga* larvae negatively impacts subsequent growth and development, which can decrease their population [20]. Despite this understanding of temperature effects on larvae, it is unclear how high temperatures impact other development stages of *B. odoriphaga* and the ecological mechanism by which high temperatures decrease the population growth. If the above assertions were made certain, we could improve the environment temperature to the suitable non-lethal temperature to limit *B. odoriphaga* development, or control their population under the level of economic damage threshold, which would decrease the damage of Chinese chives and protect the ecological balance of the species. Therefore, we designed our study to expose the pupae and unmated female and male adults of *B. odoriphaga* to different degrees of non-lethal high-temperature stress. In particular, we assessed the impact of heat stress on *B. odoriphaga* reproduction by qualitatively examining the reproductive organs of adults. Additionally, we observed the effects of non-lethal heat stress on pupae and adult growth and development, and on reproductive success (measured as the female reproductive capacity and egg hatching rate). This provides a key theoretical foundation for the development of high-temperature products for environmentally friendly pest control.

## 2. Materials and Methods

### 2.1. Bradysia odoriphaga Rearing and Maintenance

The *B. odoriphaga* population was supplied by the Institute of Vegetables and Flowers, at the Chinese Academy of Agricultural Sciences, Beijing, China. They were cultured under a constant temperature (25 ± 1 °C) in an incubator (MLR-352H-PC) (70 ± 5% RH) at a 14:10 (L:D) h photoperiod in a laboratory located at the Agricultural College of Yangtze University. Three generations of this population were raised with *A. tuberosum* rhizomes for subsequent use.

### 2.2. Impacts of High-Temperature Stress on Bradysia odoriphaga Pupae

According to our research experience, female and male pupae can be distinguished by their size: females are significantly larger than males at the same age (unpublished). Male and female pupae were separated by sex within 8 h of pupation into two clearly labeled Petri dishes. The dish bottom was covered with a 3 mm thick layer of 2% solidified agar (A109143; Aladdin, Shanghai, China), while filter paper was placed on the surface of the agar to maintain the moisture content.

#### 2.2.1. High-Temperature Impacts Growth and Development of *Bradysia odoriphaga* Pupae

A single pupa was placed on each Petri dish (*Φ* = 3 cm); 30 male and 30 female pupae were placed into the control or experimental group at a time. The experimental treatments exposed each group to 34, 37, and 40 °C temperatures for 1, 2, or 4 h. The control was exposed to 25 °C, with 30 females and 30 males for each treatment. Following the treatment, the Petri dishes were placed in an incubator at a constant temperature of 25 °C. Each treatment, as well as the control, was performed five times. The emergence of pupae was observed once a day, while the pupae were considered dead if eclosion did not begin within 10 days. Once eclosion occurred, the pupal sex was observed, while the pupae were maintained in their original Petri dishes. Adult survival was observed each day until all had died.

#### 2.2.2. Impacts of High-Temperature Stress on Egg Laying and Egg Hatching in *Bradysia odoriphaga* Pupae

Forty male and forty female pupae were placed into the control or experimental group at a time; each was placed in an individual Petri dish (*Φ* = 6 cm). The experimental treatments exposed each group to 34, 37, and 40 °C temperatures for 1, 2, or 4 h. Following the treatment, the Petri dishes were placed in an incubator at a constant temperature of 25 °C. Once emergence occurred, female and male adults were placed, one by one, into a new Petri dish within 8 h. Every six pairs were considered a replicate that were placed in an incubator at 25 °C for typical feeding. Each treatment, as well as the control, was performed five times. Adult lifespan, the number of eggs each female laid, and rate of egg hatching were all recorded.

#### 2.2.3. Impacts of High-Temperature Stress on Unmated Adult Reproductive Organs Size in *Bradysia odoriphaga* Pupae

Forty female and forty male pupae were individually placed into two Petri dishes (*Φ* = 6 cm) as a treatment or the control. Treatments were as follows: 34 °C and 37 °C for either 2 h or 4 h. The control was exposed to a constant temperature of 25 °C. After treatment, the Petri dishes were put into a constant temperature incubator at 25 °C. After pupae eclosion, the male and female adults (within 8 h of emergence) per treatment or the control were dissected, and the length of testis and ovaries was measured. Six pupae of the same sex were sampled as one replicate. Each treatment or control was repeated five times.

### 2.3. Impacts of High-Temperature Stress on the Morphology and Structure of Testis and Ovaries in Unmated Bradysia odoriphaga Adults

**Figure 1:**
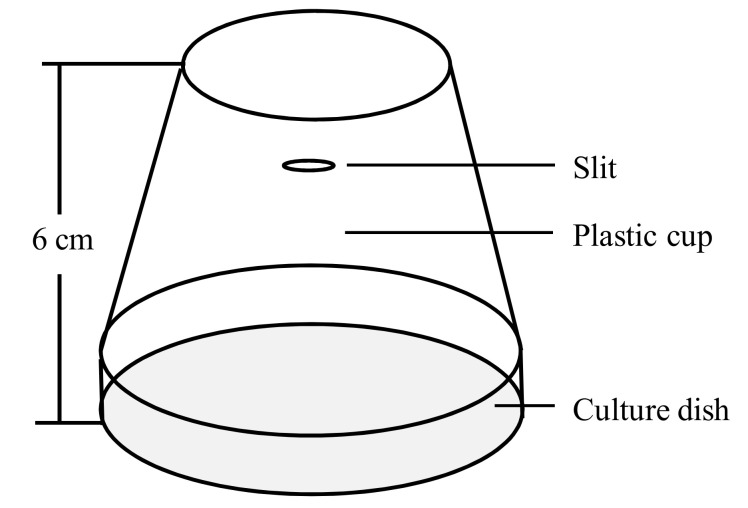
Insect cage for adults.

Forty female and forty male adults (within 8 h of emergence) were individually placed into two insect cages (Figure 1) as a treatment or the control. The treatments were as follows: exposure to 34 °C and 37 °C for either 2 h or 4 h. The control was exposed to a constant temperature of 25 °C. After treatment, the insect cages were placed into a constant temperature incubator at 25 °C to recover for 1 h or 24 h, individually. Afterward, the male and female adults were dissected and photographed, while the size of testis and ovaries was measured. Six adults of the same sex were sampled as one replicate. Each treatment or the control was repeated five times.

### 2.4. Data Analysis

Data entry was performed using Excel version 2007, while one-way analyses of variance (ANOVA) were performed using SPSS version 17.0 (SPSS Inc., Chicago, IL, USA). Data were presented as mean ± SD. Except lifetime in female and male was compared by Student’s *t*-test, other treatments were assessed by Tukey’s test. The results were considered significant when *p* < 0.05.

## 3. Results

### 3.1. Impacts of High-Temperature Stress on Bradysia odoriphaga Pupae

#### 3.1.1. Impacts of High-Temperature Stress on *Bradysia odoriphaga* Pupae Emergence Rate

**Figure 2:**
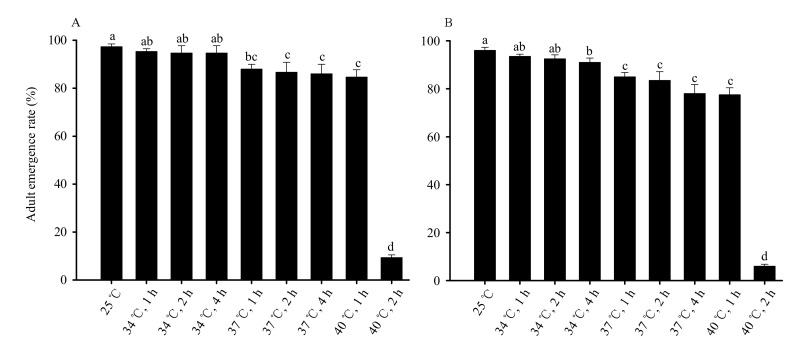
High-temperature stress affects the emergence rate in female (**A**) and male (**B**) *Bradysia odoriphaga* pupae. Data are mean ± SD of five independent replicates (n = 30 for each replicate, N = 150 treatments). Different bar labels indicate significant differences according to Tukey’s test (*p* < 0.05). Pupae for control or each treatment were placed in a single, clearly marked Petri dish. Pupal eclosion was observed daily, and pupae were considered dead if eclosion did not begin within 10 days.

The rate of emergence of the female pupae decreased as the duration and temperature of exposure increased (*F*_8,36_ = 1919.523, *p* < 0.01). The rate of emergence of female pupae when exposed to 34 °C for 1, 2, and 4 h did not significantly differ from the rate observed in the 25 °C control group. No significant differences were observed in treatments at 37 °C for 1, 2, and 4 h, and 40 °C for 1 h. The lowest rate of emergence, 9.3%, was observed in female pupae exposed to 40 °C for 2 h (Figure 2A).

The rate of emergence of the male pupae decreased as the duration and temperature of exposure increased (*F*_8,36_ = 3679.482, *p* < 0.01). The rate of emergence of male pupae exposed to 34 °C for 4 h significantly differed from the 25 °C control group. Once temperatures exceeded 37 °C, the rate of male eclosion decreased significantly below that of the 25 °C control group. No significant difference was observed among treatments at 37 °C for 1, 2, and 4 h, and 40 °C for 1 h. The lowest rate of emergence, 6.0%, was observed in male pupae exposed to 40 °C for 2 h (Figure 2B).

#### 3.1.2. Impacts of High-Temperature Stress on Adults Longevity in *Bradysia odoriphaga* Pupae

**Figure 3:**
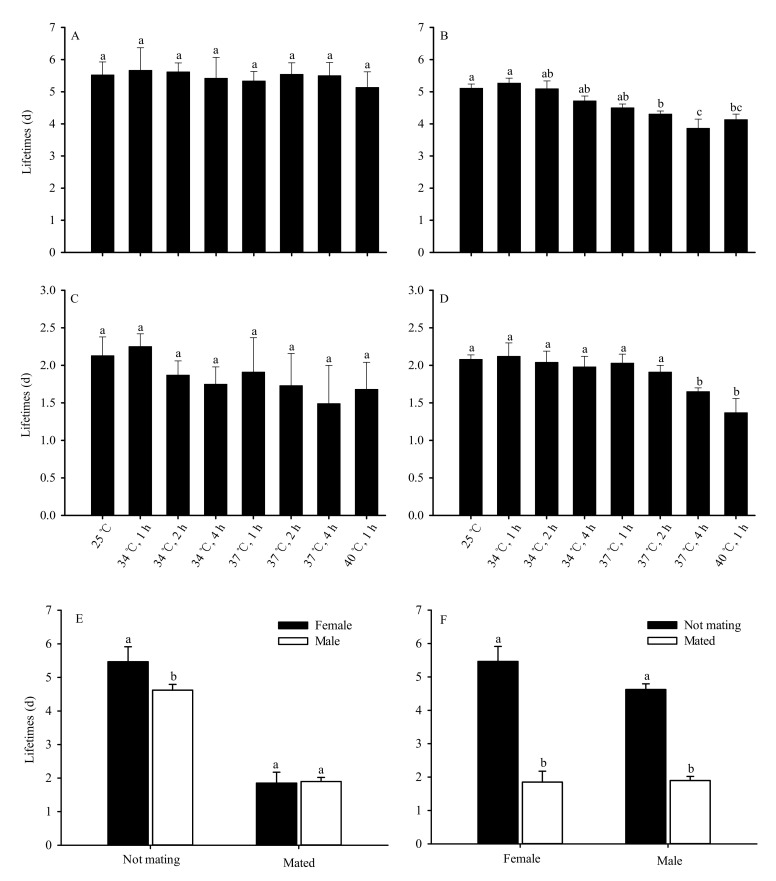
High-temperature stress impacts of adult lifespan in *Bradysia odoriphaga* pupae ((d) = days). (**A**) indicates unmated female adults; (**B**) indicates unmated male adults; (**C**) indicates mated female adults; (**D**) indicates mated male adults; (**E**) indicates comparison of lifespan between female and male adults; (**F**) indicates comparison of lifespan between mated and unmated adults. Data presented are mean ± SD of five replicates. Different bar labels in Figure (**A**–**D**) and Figure (**E**,**F**) separately indicate significant differences according to Tukey’s test and Student’s *t*-test (*p* < 0.05). Female and male adult lifespan was recorded each day once they emerged as adults. Adults were considered dead if they did not move after being touched by an object. The total number (N) of unmated female adults was 146, 143, 142, 142, 132, 130, 129, 127, and 14; the total number (N) of unmated male adults was 144, 140, 139, 137, 128, 125, 117, 116, and 9 after pupae exposure to 25 °C or 34 °C for 1 h, 34 °C for 2 h, 34 °C for 4 h, 37 °C for 1 h, 37 °C for 2 h, 37 °C for 4 h, 40 °C for 1 h, and 40 °C for 2 h, respectively. The total numbers (N) of mated female and male adults were 30 and 30, respectively, under different test conditions.

No significant differences were observed in the lifespan of unmated female adults after the pupae were subjected to various levels of short-term high-temperature stress (*F*_7,32_ = 0.451, *p* = 0.857) (Figure 3A). However, the lifespan of unmated male adults significantly decreased as the duration and temperature of exposure increased (*F*_7,32_ = 94.865, *p* < 0.01). Compared with the 25 °C control group, no significant differences were observed in the lifespan of male adults following exposure of the pupae to 34 °C for 1, 2 and 4 h, and 37 °C for 1 h. However, the lifespan of the 37 °C groups treated for 2 h and 4 h and the group treated at 40 °C for 1 h were significantly shorter than the 25 °C control group. There was no difference in male lifespan between the 37 °C group (2 h of exposure) and the 40 °C group (1 h of exposure). The shortest male lifespan, 3.86 days, occurred in the 37 °C group (4 h of exposure) (Figure 3B).

No significant differences were also observed in the lifespan of mated female adults after the pupae were subjected to various levels of short-term high-temperature stress (*F*_7,32_ = 1.174, *p* = 0.345) (Figure 3C). The lifespan of mated male adults also significantly decreased as duration and temperature of exposure increased (*F*_7,32_ 43.554, *p* < 0.01) (Figure 3D). However, the average lifespan of unmated female adults (5.47 d) significantly exceeded that of unmated male adults (4.62 d) (*t* = 11.380, *df* = 78, *p* < 0.01). The average lifespan of female adults was lower than that of male adults (1.85 days and 1.89 days respectively) after mating, though the difference was not significant (*t* = 0.272, *df* = 63.322, *p* = 0.787) (Figure 3E). The average lifespan of female (*t* = 40.474, *df* = 78, *p* < 0.01) and male (*t* = 39.138, *df* = 78, *p* < 0.01) adults significantly decreased following mating (Figure 3F).

#### 3.1.3. Impacts of High-Temperature Stress on Reproductive Capacity and Egg Hatchability in *Bradysia odoriphaga*

**Figure 4:**
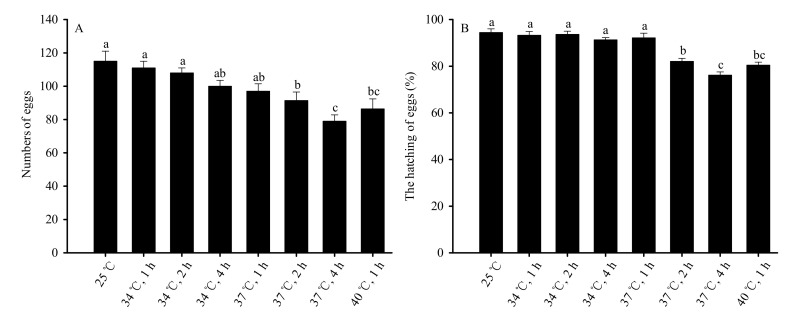
High-temperature stress impacts on reproductive capacity (**A**) and egg hatchability (**B**) for mated female adults in *Bradysia odoriphaga* pupae. Data are mean ± SD of five replicates. Different bar labels indicate significant differences according to Tukey’s test (*p* < 0.05). Female and male adults were paired and placed into different Petri dishes (*Φ* = 60 mm). One pair was placed in each Petri dish, while six pairs were considered a replicate. Microscopy was used to count the number of larvae and eggs. On the condition that some of the eggs in one Petri dish successfully hatched into larvae, the number of eggs in that dish was recorded for subsequent statistical analysis.

Female reproductive capacity was measured by the number of eggs that each female laid, and decreased significantly when the pupal duration and temperature of stress exposure increased (*F*_7,32_ = 44.586, *p* < 0.01). No significant differences were observed in the ability of mated female adults to reproduce at 34 °C of stress for 1, 2, and 4 h, or under 37 °C for 1 h when compared to the control group (25 °C). The reproductive capacity decreased significantly compared to the control group under 37 °C of treatment for 2 h and 4 h and 40 °C of treatment for 1 h. No significant difference was observed in groups exposed to 40 °C for 1 h and groups exposed to 37 °C for 2 h. The capacity of females to reproduce was the lowest following 4 h of treatment at 37 °C (Figure 4A).

High-temperature stress also significantly affected the rate of hatching (*F*_7,32_ = 114.882, *p* < 0.01). No significant difference was observed in the rate of hatching of eggs laid by mated females in the groups stressed at 34 °C for 1, 2, or 4 h, or 37 °C for 1 h compared to the control. Significant decreases were observed in groups exposed to 37 °C for 2 h and 4 h and 40 °C for 1 h compared to the control group. However, the difference between the 40 °C group (1 h of exposure) and the 37 °C group (2 h of exposure) was not significantly different. The rates of egg hatching were lowest following 4 h of stress at 37 °C (Figure 4B).

#### 3.1.4. Impacts of High-Temperature Stress on the Reproductive Organs of Unmated Adults in *Bradysia odoriphaga* Pupae

**Figure 5:**
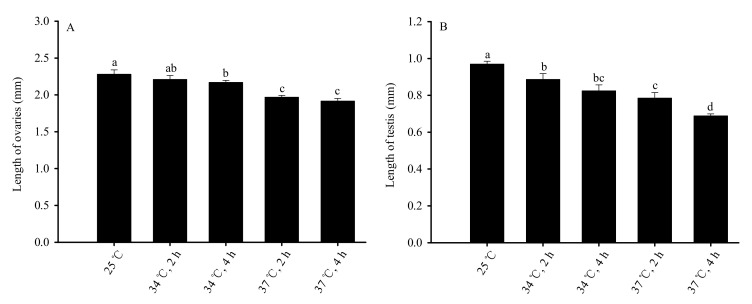
Impacts on the length of testis and ovaries of unmated male and female adults after short-term high-temperature stress on pupae. Figure (**A**) represents ovary length; Figure (**B**) represents testis length. Values are averages ± SD of five replicates. Bars with different letters are significantly difference, based on Tukey’s test (*p* < 0.05) in each panel. Male and female adults (within 8 h of eclosion) were dissected, and the length of testis and ovaries was measured. The average length of six testis or ovaries in each treatment or the control was considered a replicate and was used for statistical analysis.

Treatment of pupae with short-term high-temperature stress significantly impacted the size of the reproductive organs of the adults (Figure 5). As temperature and duration increased, ovaries length decreased (*F*_4,20_ = 40.783, *p* < 0.01). The length of ovaries of the 25 °C control group was the longest and was significantly longer than that of 34 °C for 4 h, 37 °C for 2 h and 37 °C for 4 h treatment groups. The ovaries of the treatment groups at 37 °C for 2 h and 37 °C for 4 h were the shortest (Figure 5A). Similarly, as the temperature and duration increased, the testis length decreased (*F*_4,20_ = 115.544, *p* < 0.01). The testis length was longest in the 25 °C control group and was significantly longer than that of all other treatment groups. The shortest testis was found in the group treated at 37 °C for 4 h (Figure 5B).

### 3.2. Impacts of High-Temperature Stress of Bradysia odoriphaga Unmated Adults on the Size of Reproductive Organs

**Figure 6:**
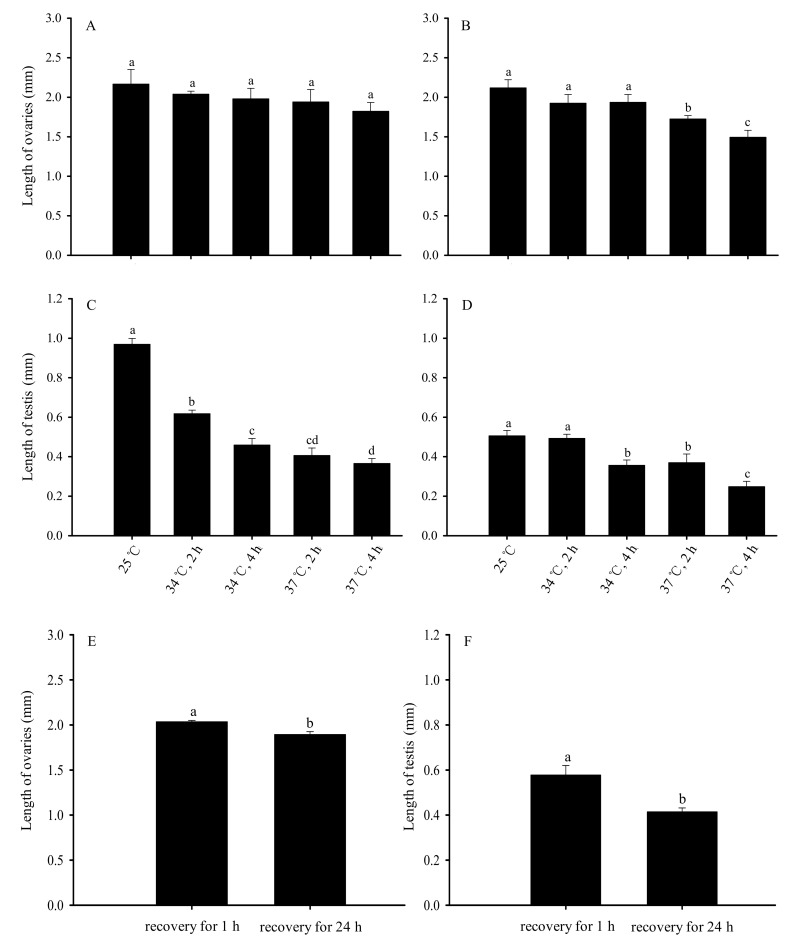
Impacts on the length of reproductive organs after short-term high-temperature stress on *Bradysia odoriphaga* unmated adults. (**A**) represent the length of the ovaries recovery for 1 h; (**B**) represent the length of the ovaries recovery for 24 h; (**C**) represent the length of the testis recovery for 1 h; (**D**) represent the length of the testis recovery for 24 h; (**E**) represent that the length of the ovaries was compared between recovery for 1 h and 24 h; (**F**) represent that the length of the testis was compared between recovery for 1 h and 24 h. Data shown are mean ± SD of five replicates. Different bar labels indicate significant differences, based on Tukey’s test (*p* < 0.05). Female adults were dissected, and the length of ovaries and testis was measured. The average length of six ovaries and six testis from each treatment was separately considered a replicate and was used for statistical analysis.

When adults were treated with short-term high-temperature stress, the size of the reproductive organs of subsequent unmated adults significantly changed (Figure 6). Compared with the ovaries of unmated female adults normally reared at 25 °C, there was no significant difference in the length of ovaries on unmated female adults after short-term high-temperature stress and 1 h recovery at 25 °C (*F*_4,20_ = 2.684, *p* = 0.062) (Figure 6A). Differing from the ovaries of unmated female adults normally reared at 25 °C, the length of the ovaries in the group that recovered at 25 °C for 24 h after short-term high-temperature treatment decreased as the temperature and duration of the stress increased (*F*_4,20_ = 43.036, *p* < 0.01). The shortest ovaries were surveyed in the group exposed to 37 °C for 4 h (Figure 6B).

Compared with the testis of unmated male adults normally reared at 25 °C, there was a significant difference in the length of testis on unmated male adults after short-term high-temperature stress and 1 h recovery at 25 °C (*F*_4,20_ = 293.858, *p* < 0.01) (Figure 6C). Additionally, under the same treatment conditions, the length of the testis of male adults after 24 h recovery was also significant difference (*F*_4,20_ = 222.723, *p* < 0.01). The shortest testis were surveyed in the group exposed to 37 °C for 4 h (Figure 6D).

Additionally, under the same treatment conditions, the length of the ovaries of female adults after 24 h recovery was significantly smaller than those of female adults after 1 h of recovery (*t* = 3.943, *df* = 35.302, *p* < 0.01) (Figure 6E). The length of the testis of male adults after 24 h recovery was also significantly smaller than those of male adults after 1 h of recovery (*t* = 3.560, *df* = 31.926, *p* < 0.01) (Figure 6F).

## 4. Discussion

The temperature of the surrounding environment is the primary factor limiting the activity, development, distribution, and reproduction of insects. High temperatures can disrupt the growth, development, and reproduction of insects [21,22]. The sensitivity of various insect species to high temperatures varies, and different insects have different physiological responses to this stress. For instance, closely related species can have significantly different diapause (*Heliothis virescent* Fabricius (Lepidoptera: Noctuidae), 32 °C [16] and *Helicoverpa armigera* Hübner (Lepidoptera: Noctuidae), 37 °C [23]), while pupae and adults of *Plutella xylostella* Linnaeus (Lepidoptera: Plutellidae) larvae exposed to 35 °C decrease in size and cannot normally reproduce [24]. Adults of *Tribolium castaneum* Herbst (Coleoptera: Tenebrionidae) cannot reproduce after being exposed to 50 °C for 39 min [25]. Insects are poikilotherms, meaning their body temperatures change rapidly as the temperature of their environment is altered, which can result in death [26]. The growth, development, and reproductive capacity can still be disrupted even if the insect is not killed. High-temperature exposure increases the preoviposition period in *Liriomyza sativae* Blanchard (Diptera: Agromyzidae), decreases the lifespan of *Laodelphax striatellus* Fallen (Hemiptera: Delphacidae) [27], decreases the ability of *Myzus persicae* Sulzer (Hemiptera: Aphididae) to reproduce [28], alters the ratio of males and females in the offspring of *Frankliniella occidentalis* Pergande (Thysanoptera: Thripidae), and alters reproduction in *Harmonia axyridis* Pallas (Coleoptera: Coccinellidae) [29]. Our results demonstrate that exposing *B. odoriphaga* pupae to heat stress (at or exceeding 37 °C) significantly lowers the rate of eclosion as the temperature and duration of exposure increase. This indicates that 37 °C is a threshold temperature for the eclosion of pupae. Shi et al. [26] determined that *B. odoriphaga* pupae were more resistant to high-temperature stresses than the other life-history stages. As high temperatures decrease the eclosion rate, other insect life stages could be affected. This study confirms that the 37 °C threshold does affect the development of pupae and the ability of adults to reproduce. This aligns with previous findings that *B. odoriphaga* populations decrease in the summertime because temperatures in certain regions of China can exceed 37 °C [19].

We determined that if pupae successfully develop into adults under heat stress, there is little effect on the lifespan of unmated female adults. However, the male lifespan decreases significantly. Liang et al. [30] demonstrated that *B. odoriphaga* males can mate several times throughout their lives. If the male lifespan decreases significantly, it will decrease the times of mating, thus reducing the population of the insect. Therefore, our study further indicated that high-temperature stress can affect the population of *B. odoriphaga* by decreasing the lifespan of males and lowering their reproductive capacity.

The lifespan of an insect is associated with its intake of energy: insects that live a long time require a lot of energy [31]. However, *B. odoriphaga* adults do not eat and have a fairly short lifespan. Male adults are significantly smaller than females, though the ability of females to fly is lower than that of males [32]. The association between energy use, energy consumption, and development would suggest that females eat more and live longer than males. This aligns with our findings. The lifespan of unmated male adults is much shorter than that of unmated female adults, though we found no significant difference in the lifespan of female and male adults following mating. This indicates that females spend more energy on reproduction and oviposition than on lifespan. The lifespan of treated females significantly decreased after they mated. Mating requires significant amounts of energy; *B. odoriphaga* males can mate several times in their lives [30]. The stress of high temperatures significantly affected male pupae and the lifespan of unmated adult males. This suggests that the restoration of reproductive and developmental functioning following stress significantly affects male lifespan. Male lifespan in *B. odoriphaga* is already shorter than female lifespan, and heat stress further widens this gap.

High-temperature stress impacts development and propagation in insects [33,34]. Our research demonstrated that female reproductive capacity and the egg hatchability of mated adult females exposed to heat stress as pupae decreased significantly as the temperature and duration increased. The reproductive capacity and rate of egg hatching were the lowest after 4 h of exposure to 37 °C. Additionally, female ovaries became shorter as the temperature and duration of heat stress increased, while the shortest ovary was found in the treatment group exposed to 37 °C for 4 h. This relationship can explain why the adult female reproductive capacity and egg hatchability both significantly decrease after the pupae are exposed to high-temperature stress. After male pupae were exposed to heat stress, the testis size in adult *B. odoriphaga* became smaller with the increase in stress temperature and duration, which may also affect the survival quality of male sperm and reduce egg hatchability [35]. The influence of high-temperature stress on the sperm survival rate of *B. odoriphaga* requires further study.

The reproductive organs of male and female *B. odoriphaga* gradually began to develop and mature during the pupal stage [28]. We found that applying non-lethal high-temperature stress to the pupae of *B. odoriphaga* impacted adult reproductive anatomy more than applying heat stress to adults. Our research also demonstrated that applying high-temperature stress to unmated female adults and providing recovery at the control temperature for 1 h had no significant effect on ovary length, while, when the recovery time increased to 24 h, the size of the ovary decreased significantly. This indicates that short-term high-temperature stress has a continuous effect on the ovaries. Research has demonstrated that female adults (*B. odoriphaga*) will discharge some of their eggs as they recover bodily functions following exposure to high-temperature stress [36]. It indicated that eggs in the ovaries could be discharged following exposure to high-temperature stress or with recovery time increased, thus decreasing the size of the ovaries. Meanwhile, unfertilized eggs discharged would indirectly decrease the population number of *B. odoriphaga*.

High temperatures adversely affect both male and female propagation [29]. Our research demonstrates that high-temperature stress on unmated *B. odoriphaga* male adults was more significant than that on unmated female adults. Male testis shrank significantly as the temperature and duration increased, and *B. odoriphaga* testis were the smallest after 4 h of exposure to 37 °C. Moreover, under the same treatment temperature and duration, the testis of male adults that were allowed to recover for 24 h were significantly smaller than the testis of male adults that were allowed to recover for 1 h. Previous research has demonstrated that high temperatures can cause *Grapholitha molesta* Busck (Lepidoptera: Tortricidae) to expel semen from the testis, thereby affecting the size of the testis [37]. Consequently, we suspect that high temperatures impact the formation of male testis, including the potential loss of sperm count or motility, thus indirectly decreasing the population number of *B. odoriphaga*. Certainly, the specific effects of high-temperature on the formation and activity of *B. odoriphaga* testis requires further study.

## 5. Conclusions

We found that *B. odoriphaga* pupae exposed to heat stress exhibit greater constraints on growth and development, female reproductive capacity, hatchability, and testis and ovary size than adults. We also found that high-temperature stress impacts males more than females. Moreover, our results demonstrate that 37 °C is a crucial temperature for controlling populations of *B. odoriphaga*. Altogether, these results demonstrate that heat stress can be used to negatively affect *B. odoriphaga*, providing an environmentally friendly control strategy for an economically devastating pest.

## Data Availability

The data presented in this study are available in this article.
